# Nail Isthmus: A Distinct Region of the Nail Apparatus

**DOI:** 10.1155/2012/925023

**Published:** 2012-02-09

**Authors:** Naoki Oiso, Ichiro Kurokawa, Akira Kawada

**Affiliations:** ^1^Department of Dermatology, Kinki University Faculty of Medicine, Osaka-Sayama 589-8511, Japan; ^2^Department of Dermatology, Acne Clinical and Research Center, Meiwa Hospital, Nishinomiya 663-8186, Japan

## Abstract

The nail unit is constructed by distinctly regulated components. The nail isthmus is a lately proposed region as a transitional zone between the most distal part of the nail bed and the hyponychium. It is difficult to recognize the nail isthmus in the normal nail, but it is easy to identify the region in nail disorders such as pterygium inversum unguis and ectopic nail. We describe structure and putative function of the nail isthmus via histopathologic features of pterygium inversum unguis and ectopic nail.

## 1. The Nail Isthmus

**Figure 1:**
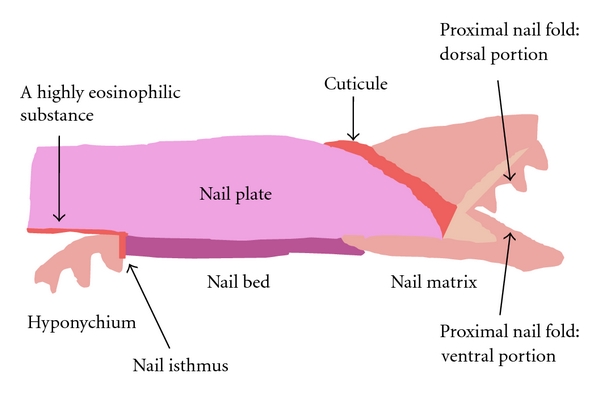
A diagram of the nail apparatus. The nail isthmus is present in the transitional zone between the most digital part of the nail bed and the hyponychium.

The nail unit has distinct structure. The concept of the nail isthmus was recently proposed by Perrin in 2007 [1]. The region is present in the transitional zone between the most digital part of the nail bed and the hyponychium (Figure 1) [1–3]. Perrin described the four typical features of the nail isthmus: (i) the maintenance of the longitudinal ridge pattern of the nail bed, (ii) a discontinuous and thin granular layer, (iii) a peculiar and thin compartment of pale and nucleated corneocytes, and (iv) a profile of transitional keratin expression [1–3]. The nail isthmus is almost invisible in the normal nail, but it is able to be identified in nail disorders such as pterygium inversum unguis [4] and ectopic nail [5].

## 2. The Structure of the Nail Isthmus

**Figure 2:**
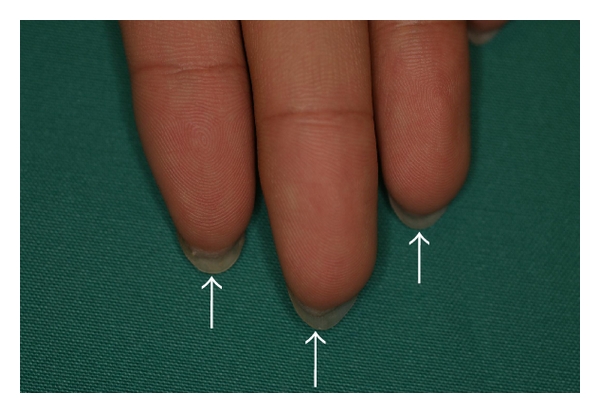
A ventral appearance of pterygium inversum unguis in a 16-year-old Japanese man. Marked subungual keratotic thickening is present on the distal nail unit of the left fingers (arrows).

**Figure 3:**
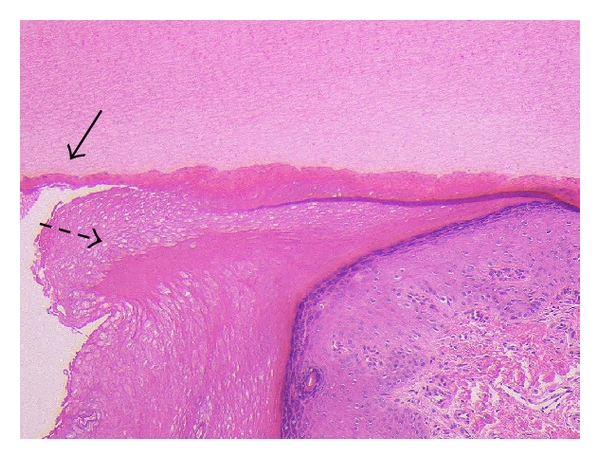
A biopsy specimen from the left second finger showed (i) a marked, highly eosinophilic, keratinized substance attaching the distal and visceral nail plate (arrow) and (ii) a whorled, highly eosinophilic, keratinized substance into the horny layer of the finger tip (dotted arrow) (Hematoxylin and eosin staining, original magnification ×100).

**Figure 4:**
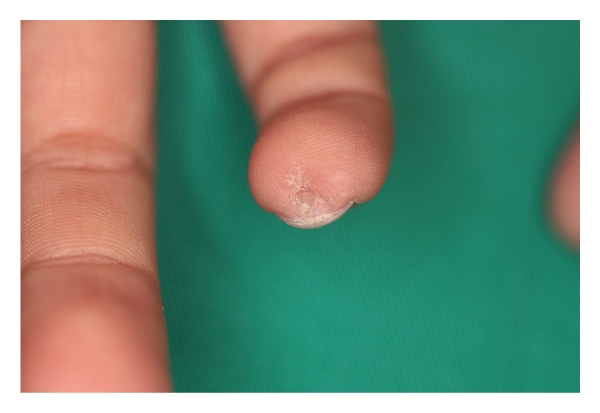
An appearance of an ectopic nail in a 7-year-old Japanese man.

**Figure 5:**
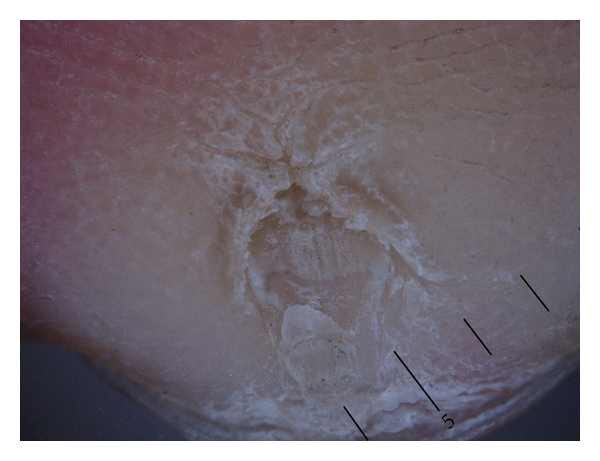
A dermoscopic figure of the ectopic nail. Most of the nail plate was covered by cuticle.

The nail isthmus is composed of two distinct parts. A histopathological study of the nail isthmus with a case of pterygium inversum unguis identified two substances: (i) a marked, highly eosinophilic, keratinized substance attaching the distal and visceral nail plate and (ii) a whorled, highly eosinophilic, keratinized substance into the horny layer of the finger tip (Figures 2 and 3) [4]. The former substance is constructed by an extraordinary mode of keratinization with a compartment of pale and nucleated corneocytes [1, 4]. Another histopathological study with a case of ectopic nail (Figures 4 and 5) showed two distinct parts: (i) a proximal and narrow part anchored to the nail plate and (ii) a distal and wide part constructed with highly eosinophilic structure [5].

The highly eosinophilic structures are identical to semihard keratin. The nail apparatus is sequentially composed of soft keratin in the proximal nail fold, semihard keratin in the cuticle, hard keratin in the nail plate from the nail matrix and the nail bed, semihard keratin in the nail isthmus, and soft keratin in the hyponychium (Figure 1).

**Figure 6:**
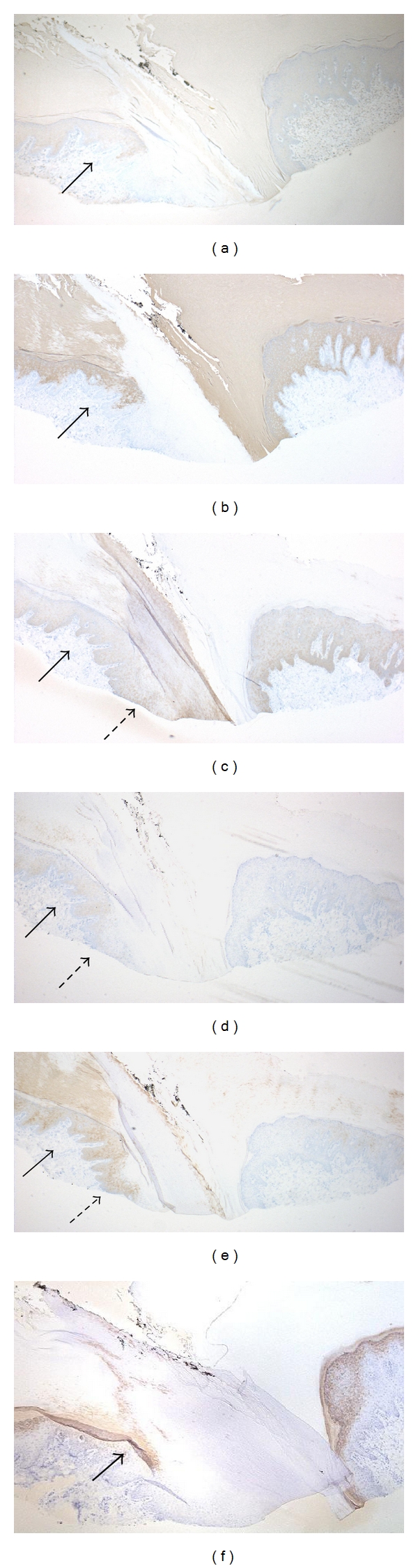
(a) K1 was positively stained in the suprabasal layers of the nail isthmus (arrow) (original magnification ×40). (b) K10 was expressed in the suprabasal layers of the nail isthmus (arrow) (original magnification ×40). (c) K14 was shown in the whole layers of the nail bed (dotted arrow) and nail isthmus (arrow) (original magnification ×40). (d) K16 was slightly detected in the suprabasal layers of the nail bed (dotted arrow) and nail isthmus (arrow) (original magnification ×40). (e) K17 was considerably expressed in the suprabasal layers of the nail bed (dotted arrow) and nail isthmus (arrow) (original magnification ×40). (f) Filaggrin was identified in the granular layer of the nail isthmus (arrow) (original magnification ×40).

Immunohistochemical study of the regional keratin and filaggrin in a case of ectopic nail showed keratin 1 (K1) and K10 expression in the suprabasal layers of the nail isthmus (Figures 6(a) and 6(b)), K14 in all of the layers in the nail bed and the nail isthmus (Figure 6(c)), K16 and K17 in the suprabasal layers of the nail bed and the nail isthmus (Figures 6(d) and 6(e)), and filaggrin in the granular layer of the nail isthmus (Figure 6(f)) [5]. This result was consistent with the previous immunohistochemical study by Perrin [2]. Keratins are heteropolymeric filaments formed by pairing type I (acidic) and type II (basic to neutral) keratins [6]. Perrin's and our results indicate that the pair of K5/K14 are expressed in the whole keratinocytes in the nail bed, the nail isthmus, and the hyponychium, K6/K16 and K17 are in the suprabasal keratinocytes in the nail bed and the nail isthmus, and K1/K10 are in the suprabasal keratinocytes in the nail isthmus and the hyponychium. Thus, the nail isthmus has both features of keratin expression (producing semi-hard keratin) in the nail bed (producing hard keratin) and the hyponychium (producing soft keratin). The filaggrin expression indicates the truncation of onychokeratinization in the nail bed and the emergence of specific keratinization producing nucleated corneocytes via the granular layer in the nail isthmus.

## 3. The Function of the Nail Isthmus

The nail isthmus showed two regions; a proximal and narrow part and a distal and wide part. The proximal and narrow region has supposed function as an anchor for the inferior border of the nail plate. The distal and wide region produces semihard keratins possibly against repeated trauma toward the separated area between the nail plate and the hyponychium. Pterygium inversum unguis may occur after a cerebral vascular event resulted in hemi-paralysis [7]. Ectopic nail has no function as a nail. The aberrant keratinization of the nail isthmus in pterygium inversum unguis and ectopic nail might not be caused by overproduction of semihard keratin but by excess persistent presence of semihard keratin because of little or no traumas toward the region.

## 4. Conclusion

The nail isthmus expresses a profile of transitional keratins and is probably constructed by two regions. One is a proximal region producing a marked, highly eosinophilic, keratinized substance attaching the distal and ventral nail plate. The region produces a peculiar and thin compartment of pale and nucleated corneocytes via the granular layer and probably maintains the longitudinal ridge pattern of the nail bed. Another is the distal region producing a whorled, highly eosinophilic, keratinized substance and may protect the binding between the nail plate and the proximal nail isthmus from repeated trauma.

 The nail isthmus is one of the distinctly regulated regions of the nail apparatus. A recent proposal of the nail isthmus brings us to reevaluate the pathogenesis of the nail disorders. In the future, further study will elucidate more precise structure and function of the nail isthmus.

## References

[1] Perrin C (2007). Peculiar zone of the distal nail unit: the nail isthmus. *American Journal of Dermatopathology*.

[2] Perrin C (2007). Expression of follicular sheath keratins in the normal nail with special reference to the morphological analysis of the distal nail unit. *American Journal of Dermatopathology*.

[3] Perrin C (2008). The 2 clinical subbands of the distal nail unit and the nail Isthmus. Anatomical explanation and new physiological observations in relation to the nail growth. *American Journal of Dermatopathology*.

[4] Oiso N, Narita T, Tsuruta D, Kawara S, Kawada A (2009). Pterygium inversum unguis: aberrantly regulated keratinization in the nail isthmus. *Clinical and Experimental Dermatology*.

[5] Oiso N, Kurokawa I, Tsuruta D (2011). The histopathological feature of the nail isthmus in an ectopic nail. * American Journal of Dermatopathology*.

[6] Moll R, Divo M, Langbein L (2008). The human keratins: biology and pathology. *Histochemistry and Cell Biology*.

[7] Vadmal M, Reyter I, Oshtory S, Hensley B, Woodley DT (2005). Pterygium inversum unguis associated with stroke. *Journal of the American Academy of Dermatology*.

